# Nerves are more abundant than blood vessels in the degenerate human intervertebral disc

**DOI:** 10.1186/s13075-015-0889-6

**Published:** 2015-12-21

**Authors:** Abbie L. A. Binch, Ashley A. Cole, Lee M. Breakwell, Antony L. R. Michael, Neil Chiverton, Laura B. Creemers, Alison K. Cross, Christine L. Le Maitre

**Affiliations:** Biomolecular Sciences Research Centre, Sheffield Hallam University, Howard Street, Sheffield, S1 1WB UK; Department of Spinal Surgery, Sheffield Teaching Hospitals, Sheffield, UK; Department of Orthopaedics, University Medical Centre Utrecht, Utrecht, The Netherlands

**Keywords:** Intervertebral disc, Nucleus pulposus, Nerves, Blood vessels

## Abstract

**Background:**

Chronic low back pain (LBP) is the most common cause of disability worldwide. New ideas surrounding LBP are emerging that are based on interactions between mechanical, biological and chemical influences on the human IVD. The degenerate IVD is proposed to be innervated by sensory nerve fibres and vascularised by blood vessels, and it is speculated to contribute to pain sensation. However, the incidence of nerve and blood vessel ingrowth, as well as whether these features are always associated, is unknown. We investigated the presence of nerves and blood vessels in the nucleus pulposus (NP) of the IVD in a large population of human discs.

**Methods:**

Immunohistochemistry was performed with 61 human IVD samples, to identify and localise nerves (neurofilament 200 [NF200]/protein gene product 9.5) and blood vessels (CD31) within different regions of the IVD.

**Results:**

Immunopositivity for NF200 was identified within all regions of the IVD within post-mortem tissues. Nerves were seen to protrude across lamellar ridges and through matrix towards NP cells. Nerves were identified deep within the NP and were in many cases, but not always, seen in close proximity to fissures or in areas where decreased matrix was seen. Fifteen percent of samples were degenerate and negative for nerves and blood vessels, whilst 16 % of all samples were degenerate with nerves and blood vessels. We identified 52 % of samples that were degenerate with nerves but no blood vessels. Interestingly, only 4 % ofall samples were degenerate with no nerves but positive for blood vessels. Of the 85 samples investigated, only 6 % of samples were non-degenerate without nerves and blood vessels and 7 % had nerves but no blood vessels.

**Conclusions:**

This study addresses the controversial topic of nerve and blood vessel ingrowth into the IVD in a large number of human samples. Our findings demonstrate that nerves are present within a large proportion of NP samples from degenerate IVDs. This study shows a possible link between nerve ingrowth and degeneration of the IVD and suggests that nerves can migrate in the absence of blood vessels.

## Background

Chronic low back pain (LBP) is the most common cause of disability worldwide [1], affecting 80 % of the population at some point during life. It is estimated that 40 % of these cases are attributable to intervertebral disc (IVD) degeneration [2]. New ideas surrounding LBP are slowly emerging that are based on studies demonstrating interactions between mechanical, biological and chemical influences on the human IVD.

LBP can be classified as specific or non-specific. Specific back pain can be induced by trauma, spinal tumours or infection. However, non-specific back pain, where the root cause is unidentifiable, occurs in 80–90 % of LBP cases [3]. Studies using animal models to identify the innervation patterns of the lumbar discs have revealed that the dorsal portion of L5-S1 is innervated by the dorsal root ganglion (DRG) from L2 by the paravertebral trunk, whereas L3-L5 DRG innervate through the sinuvertebral nerve, hence these patients feel non-specific pain [4, 5].

The IVD is considered the largest aneural structure within the human body and is composed of three main anatomical regions: the central nucleus pulposus (NP), which is constrained by the annulus fibrosus (AF) and the cartilaginous endplate (CEP). The IVD is a poorly innervated organ, with sensory and sympathetic perivascular nerve fibres penetrating approximately 3 mm into the outer three lamellae of the AF [6–8]. Nerve fibres seen within the normal IVD are typically very fine in diameter, suggesting that they are C-fibres [9], which typically contain neurotransmitters. Substance P and calcitonin gene-related protein (CGRP) are known to be involved in nociception.

Although back pain is strongly associated with nerve root compression due to impingement of lumbar IVDs, chronic LBP is suggested to be exacerbated by the ingrowth of these peptides containing sensory nerve fibres into the deeper layers of the lumbar IVD. How these nerves are able to enter the usually aneural tissue is yet to be fully elucidated. Recent studies have demonstrated the ability of NP cells to increase their expression of neurotrophins and angiogenic factors in response to various factors, such as mechanical injury [10, 11], strain [11] and pro-inflammatory cytokines [12–16]. The expression of such molecules is suggested to potentiate the survival and migration of nerve and blood vessels into the degenerate IVD. In the healthy adult IVD, a number of repulsive factors exist which prevent nerve and endothelial cell ingrowth: aggrecan [17, 18]; chondromodulin [19] and, more recently, the semaphorins [20]. However, as expected, during degeneration these factors are reduced, allowing unrestricted access to the IVD.

In 1997, Freemont and colleagues used immunohistochemistry to investigate the localisation of nerve and blood vessels within the deeper layers of the IVD and documented that these nerves contained sensory peptides [21]. This study was followed up by many groups hoping to identify a route in which these nerves and blood vessels enter the IVD. Many have come to the consensus that annular tears or fissures may provide a source of entry. Stefanakis et al. showed prominence in nerve and blood vessel localisation to fissures. They demonstrated that these fissures have a lowered fluidic pressure, thus making them favourable for the growth and survival of nerves and blood vessels because in the healthy IVD the swelling pressure exhibited by the NP causes the collapse of such cells and prevents the occurrence of blood vessels [22].

The mechanisms which promote neoinnervation and neovascularisation are yet to be fully understood and are a topic of interest in association with their role in pain generation. The aim of this study was to identify and localise nerve and blood vessels within human IVD tissue obtained from surgery and post-mortem (PM) to test the hypothesis that nerve and blood vessels are present within the deeper layers of the degenerate human IVD.

## Material and methods

### Human tissue

Human IVD tissue was obtained from discectomy surgery for nerve root compression or from PM examination (processed within 72 h after death) with informed consent of the patient or the patient’s relatives. Ethical approval was obtained from Sheffield Research Ethics Committee (09/H1308/70) for 61 surgical IVD samples from 61 individuals, for 4 PM samples from 2 individuals, and for 20 PM samples from the Department of Pathology of the University Medical Centre (UMC), Utrecht Biobank, UMC Utrecht, used in line with the code of proper secondary use of human tissue (Table 1). Surgical samples were classified as intact or non-intact at surgery. Intact samples were obtained from discs where the AF remained intact (i.e., discs remained contained), whilst in non-intact samples the AF was ruptured and NP material was either extruded or sequestered.

### Tissue processing

Tissue processed at Sheffield Hallam University consisting of AF and NP was fixed in 10 % neutral buffered formalin and processed to paraffin wax. Tissue processed at Utrecht was obtained 24 h after patient death, and IVD slices were decalcified in Kristensen’s solution (50 % formic acid and 68 g/L sodium formate) in a microwave oven at 150 W and 50 °C for 6 h as previously described [23, 24]. Sections were dehydrated and rinsed in xylene before being embedded in paraffin wax. Following embedding, 4-μm sections were received from Utrecht University, and 10-μm sections of the surgical samples were cut and histologically graded using previously published criteria [13, 25]. Briefly, sections were scored numerically between 0 and 12 on the basis of presence of cell clusters, fissures, loss of demarcation and haematoxophilia (indicating reduced proteoglycan content). A score of 0–3 indicates a histologically normal (non-degenerate) IVD, and a grade ≥4 indicates evidence of degeneration.

### Immunohistochemistry

Immunohistochemistry was used to investigate and identify neurofilament 200 (NF200, a nerve marker), protein gene product 9.5 (PGP9.5, a nerve marker) and cluster of differentiation factor 31 (CD31, a blood vessel marker) immunopositivity within rat spinal cord as a positive control obtained from Charles River Laboratories (Harlow, UK), as well as human IVD tissues, as previously described [26]. To visualise the morphology and staining patterns of nerves stained with either NF200 or PGP9.5, different thicknesses of rat spinal cord were sectioned, which enabled the selection of optimal tissue thickness to take forward for human IVD samples. Briefly, 4-, 10- or 20-μm sections of rat spinal cord tissue or 4- or 10-μm sections of IVD samples were dewaxed, rehydrated and blocked with endogenous peroxidase. Antigen retrieval methods were used (Table 2). Following Tris-buffered saline (TBS) washes, non-specific binding sites were blocked at room temperature for 2 h with either 25 % wt/vol goat or rabbit serum (Abcam, Cambridge, UK) in 1 % wt/vol bovine serum albumin (BSA; Sigma-Aldrich, Poole, UK) in TBS (Table 2). Sections were incubated overnight at 4 °C with either mouse or rabbit polyclonal antibodies (Table 2). Negative controls in which rabbit and mouse immunoglobulin G (Abcam) replaced the primary antibody at an equal protein concentration were used. Slides were washed in TBS, and a 1:500 dilution of 1 % wt/vol BSA/TBS biotinylated secondary antibody was applied for 30 min at room temperature (Table 1). Binding of the secondary antibody was detected using a streptavidin–biotin complex (Vector Laboratories, Peterborough, UK) technique with 0.08 % vol/vol hydrogen peroxide in 0.65 mg/ml 3,3′-diaminobenzidine tetrahydrochloride (Sigma-Aldrich) in TBS. Sections were counterstained with Mayer’s haematoxylin (Leica Microsystems, Newcastle upon Tyne, UK), dehydrated, cleared and mounted with Pertex mounting medium (Leica Microsystems).

**Table 1:** Patient details of human IVD samples

Reference ID		Source	IVD level	IVD intact?	Histological grade	Classification	Nerves identified in abnormal location	Blood vessels identified in abnormal location
HD	31	PM (SHU)	L3-L4	Yes	3.0	ND	Yes	No
HD	32	PM (SHU)	L3-L4	Yes	4.5	D	Yes	No
HD	37	PM (SHU)	L3-L4	Yes	3.0	ND	Yes	No
HD	38	PM (SHU)	L4-L5	Yes	7.5	D	Yes	No
HD	39	PM (SHU)	L5-S1	Yes	11.5	D	Yes	Yes
HD	E13-00073	PM (UT)	L4-L5		5	D	Yes	No
HD	506-290	PM (UT)	L4-L5		9	D	Yes	No
HD	507-280	PM (UT)	L4-L5		7	D	Yes	Yes
HD	506-272	PM (UT)	L4-L5		10	D	Yes	No
HD	508-263	PM (UT)	L4-L5		11	D	Yes	No
HD	507-234	PM (UT)	L4-L5		6	D	Yes	No
HD	508-223	PM (UT)	L4-L5		7	D	No	No
HD	506-219	PM (UT)	L4-L5		7	D	No	No
HD	506-207	PM (UT)	L4-L5		7	D	Yes	No
HD	506-191	PM (UT)	L4-L5		11	D	Yes	No
HD	507-192	PM (UT)	L4-L5		11	D	Yes	Yes
HD	506-179	PM (UT)	L4-L5		11	D	Yes	Yes
HD	507-164	PM (UT)	L4-L5		5	D	No	No
HD	506-145	PM (UT)	L4-L5		7	D	Yes	No
HD	506-136	PM (UT)	L4-L5		9	D	Yes	Yes
HD	506-134	PM (UT)	L4-L5		11	D	Yes	Yes
HD	507-110	PM (UT)	L4-L5		8	D	Yes	No
HD	507-069	PM (UT)	L4-L5		11	D	No	No
HD	508-070	PM (UT)	L4-L5		9	D	No	Yes
HD	507-066	PM (UT)	L4-L5		12	D	Yes	No
HD	507-051	PM (UT)	L4-L5		10	D	Yes	Yes
HD	507-001	PM (UT)	L4-L5		6	D	Yes	No
HD	1	Surgical	L4-L5	No	2.5	ND	No	No
HD	5	Surgical	L5-S1	Yes	8.5	D	No	No
HD	19	Surgical	L5-S1	No	9.0	D	Yes	No
HD	46	Surgical	L5-S1	Yes	8.5	D	Yes	No
HD	53	Surgical	L5-S1	No	7.0	D	Yes	Yes
HD	55	Surgical	L5-S1	No	6.0	D	Yes	Yes
HD	58	Surgical	L5-S1	No	8.0	D	Yes	No
HD	60	Surgical	L5-S1	Yes	5.0	D	Yes	Yes
HD	66	Surgical	L3-L4	Yes	9.0	D	Yes	No
HD	67	Surgical	L4-L5	Yes	5.0	D	No	No
HD	68	Surgical	L4-L5	Yes	9.0	D	Yes	Yes
HD	69	Surgical	L5-S1	No	8.5	D	Yes	No
HD	70	Surgical	L5-S1	Yes	7.5	D	No	Yes
HD	71	Surgical	L5-S1	Yes	6.5	D	Yes	No
HD	72	Surgical	C5-C6	Yes	9.5	D	Yes	No
HD	74	Surgical	L4-L5	No	7.5	D	No	No
HD	78	Surgical	C3-C4	Yes	10.0	D	Yes	No
HD	84	Surgical	C6-C7	Yes	5.0	D	Yes	No
HD	85	Surgical	L2-L3	No	7.0	D	Yes	Yes
HD	88	Surgical			6.5	D	Yes	No
HD	89	Surgical	L5-S1	No	4	ND	Yes	No
HD	90	Surgical			11.0	D	Yes	No
HD	91	Surgical			6	D	Yes	No
HD	92	Surgical	L5-S1		3.5	ND	Yes	No
HD	93	Surgical	L5-S1		7.0	D	Yes	No
HD	94	Surgical	L5-S1	Yes	6.0	D	Yes	No
HD	95	Surgical	L5-S1	Yes	6.0	D	Yes	Yes
HD	96	Surgical	C5-C6	No	9.0	D	Yes	No
HD	98	Surgical	L3-L4	Yes	6.0	D	Yes	No
HD	101	Surgical	C6-C7	No	4.5	D	Yes	No
HD	104	Surgical	L5-S1	No	5.5	D	Yes	No
HD	106	Surgical	L4-L5	Yes	5.0	D	Yes	No
HD	110	Surgical	L3	No	3.0	ND	No	No
HD	112	Surgical	L5-S1	No	11.0	D	No	No
HD	124	Surgical	L4-L5	No	3.5	ND	No	No
HD	127	Surgical	L5-S1	No	4.0	ND	Yes	No
HD	146	Surgical	L5-S1		5.85	D	Yes	No
HD	158	Surgical	L4-L5	No	7.0	D	Yes	No
HD	159	Surgical	L4-L5	No	3.0	ND	Yes	No
HD	161	Surgical	C6-C7	Yes	7.0	D	Yes	No
HD	170	Surgical	L5-S1	No	8.0	D	Yes	No
HD	174	Surgical	L5-S1	Yes	7.0	D	Yes	No
HD	184	Surgical	L4-L5		8.0	D	Yes	No
HD	207	Surgical	L5-S1	No	7.0	D	Yes	No
HD	225	Surgical	L5-S1	Yes	9.0	D	No	No
HD	226	Surgical	L3-L4	No	5.5	D	Yes	Yes
HD	230	Surgical	L5-S1	No	8.5	D	Yes	No
HD	231	Surgical	L4-L5	No	5.0	D	No	NO
HD	233	Surgical	L5-S1	Yes	9.5	D	Yes	No
HD	234	Surgical	L5-S1	Yes	8.5	D	Yes	No
HD	243	Surgical	L4-L5	Yes	6.0	D	Yes	Yes
HD	245	Surgical	L4-L5	Yes	4.0	ND	No	No
HD	253	Surgical	C5-C6	Yes	5.0	D	Yes	No
HD	263	Surgical			9.0	D	No	Yes
HD	272	Surgical	L5-S1	No	4.5	D	Yes	No
HD	276	Surgical	C5-C6		6.0	D	No	No

*PM* post-mortem, *IVD* intervertebral disc, *SHU* were processed at Sheffield Hallam University, *UT* were processed in Utrecht, *L* lumbar, *C* cervical, *ND* non-degenerate, *D* degenerate

**Table 2:** Antibodies used for immunohistochemistry

Target antibody	Clonality	Optimal dilution	Antigen retrieval method	Secondary antibody	Serum block
NF200 (ab82259)	Mouse monoclonal	1:400	Heat	Rabbit anti-mouse	Rabbit
PGP9.5 (ab8189)	Mouse monoclonal	1:200	Heat	Rabbit anti-mouse	Rabbit
CD31 (ab28364)	Rabbit polyclonal	1:400	Enzyme	Goat anti-rabbit	Goat

*CD31* cluster of differentiation factor 31, *NF200* neurofilament 200, *PGP* protein gene product

All slides were visualised using a BX60 microscope (Olympus, Southend-on-Sea, UK), and images were captured using a digital camera and QCapture Pro v8.0 software (MediaCybernetics, Marlow, UK). Tissue samples were investigated for expression of NF200-immunopositive nerve cells and CD31-immunopositive blood vessels within the NP and inner annulus fibrosus (IAF) (where present). Within PM tissues where outer AF and CEP were present, nerves and blood vessels in these normal locations were not recorded as abnormal nerve and blood vessel presence.

### Statistical analysis

Firstly, a *χ*^2^ test and Fisher’s exact test were used to identify any differences between degenerate and non-degenerate samples with or without nerves and blood vessels. Following this step, a test of two independent proportions was used to identify significance between the degenerate and non-degenerate groups. Paired proportion tests were used to identify differences between proportions of nerve and blood vessels within the non-degenerate and degenerate groups.

## Results

### Rat spinal cord optimisation of two nerve markers

**Fig. 1:**
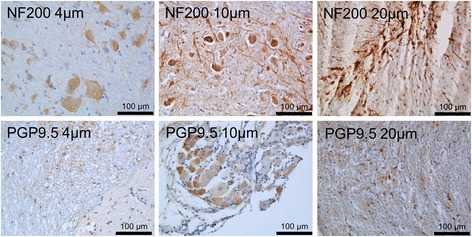
Rat spinal cord optimisation of neurofilament 200 (NF200) and protein gene product 9.5 (PGP9.5) staining at different sectioning thicknesses

Rat spinal cord was commercially obtained to optimise the staining techniques and determine the ideal tissue thickness to positively identify NF200- and PGP9.5-positive staining patterns. Varying thicknesses of tissue were sectioned, and different staining patterns were observed for NF200 and PGP9.5 (Fig. 1). Staining of tissues at a thickness of 4 μm for NF200 was very clear within the cell body, but axonal staining was lost due to the thickness (Fig. 1). PGP9.5 was unclear at 4 μm. Tissues sectioned at 20 μm and stained with NF200 demonstrated filamentous staining tracking along the matrix (Fig. 1). Staining patterns observed for PGP9.5 immunopositivity at 20-μm thickness were localised to the cell body, but this staining was not as obvious as the staining on a 10-μm section (Fig. 1). On the basis of this result, it was concluded that 10-μm sections would be taken forward for IVD visualisation of nerve and blood vessels within human surgical samples.

### Identification of nerve and blood vessels in human IVD tissues

**Fig. 2:**
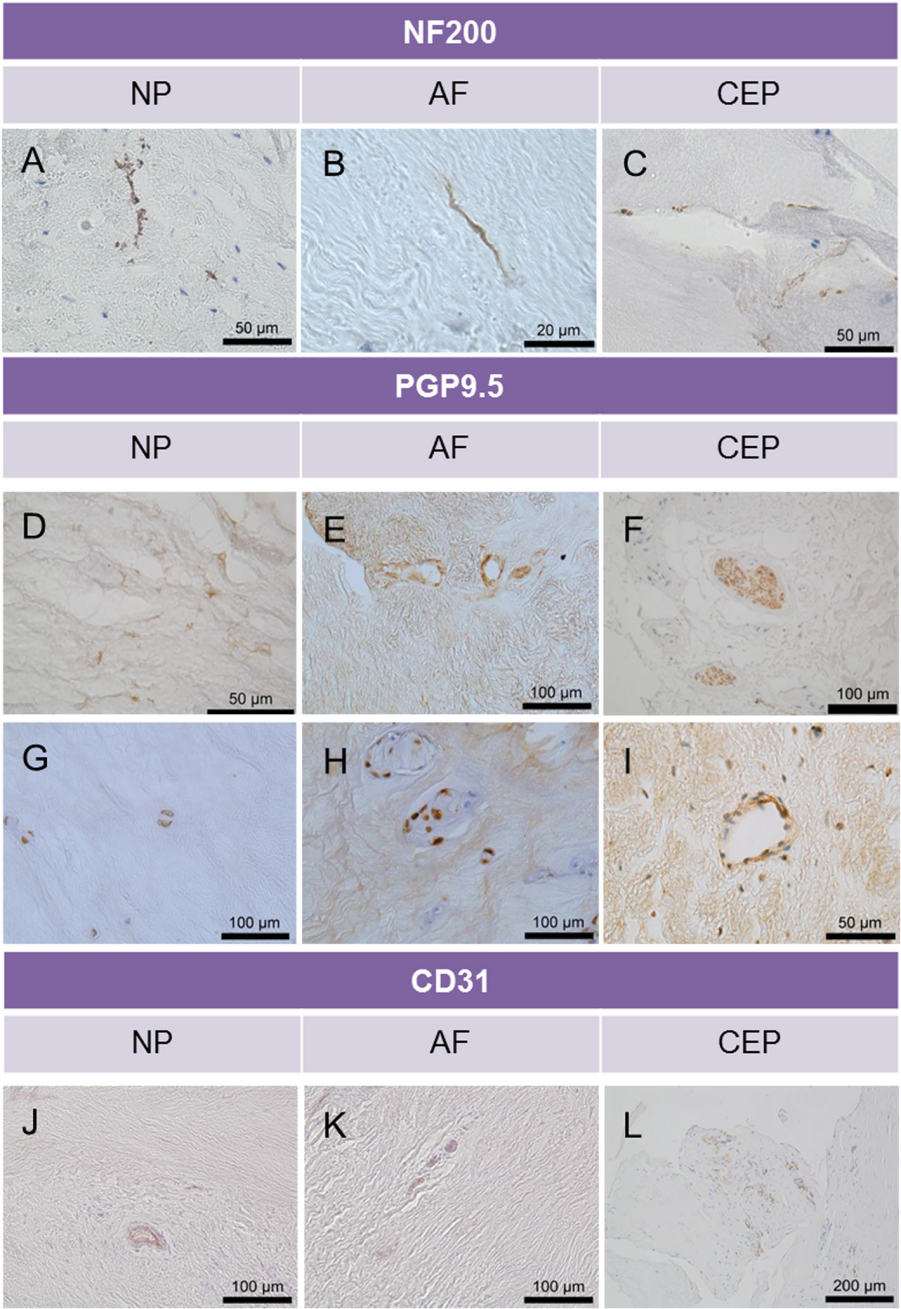
Expression of neurofilament 200 (NF200), protein gene product 9.5 (PGP9.5; (nerve) and cluster of differentiation factor 31 (CD31; endothelial) immunopositive tissues within human intervertebral disc (IVD) samples. NF200-positive tissue demonstrates strong staining of the axon and dendrites (**a** and **b**). Nerves were seen tracking along fissures (**c**). PGP9.5 staining revealed neuronal staining following the tracks of the matrix in the nucleus pulposus (NP) (**d**), surrounding what appear to be blood vessels (**e**–**i**) and nerve bundles within the cartilaginous endplate (CEP) (**f**). Cells within the NP (**g**) and annulus fibrosus (AF) (**h**) were positive for PGP9.5. Blood vessels were evident along the transition zone between AF and NP (**j**), the outer annulus fibrosus (**k**) and also within regions above the CEP (**l**)

Immunopositivity for NF200 was identified within the outer annulus fibrosus (OAF), IAF and NP of IVDs with varying grades of degeneration (Fig. 2a). In many cases, immunopositive nervous tissue followed the tracks of the IVD lamella (Fig. 2b) or fissures (Fig. 2c), but in some cases nerves were seen to protrude across the lamellar ridges or extracellular matrix (Fig. 2a) within the NP towards NP cells.

Nerves were identified deep within the NP of PM and surgical samples and were in many cases, but not always, seen in close proximity to fissures (Fig. 2c, e) or in areas where decreased matrix was seen.

The morphology of the nerves varied, depending on the orientation of the nerves in the section. For example, in Fig. 2a and b, nerve fibres and processes are visible, whereas in Fig. 2f a cross-section of tissue reveals a bundle of nerves stained with PGP9.5. Immunopositivity for PGP9.5 varied throughout the sample cohort. A finding of particular interest was the immunopositivity of NP cells themselves in a large group of herniated tissue (Fig. 2g, h). In some cases, nerves were found next to blood vessels within the IAF (Fig. 2e) and also in close proximity to infiltrating cells in herniated samples, which are thought to be immune in origin. Blood vessels were identified on serial haematoxylin and eosin–stained sections and further confirmed by immunohistochemistry with detection of CD31 (Fig. 2j–l). On the basis of this investigation, NF200 was taken forward as a marker of choice for the identification of nerves due to the specificity within the NP tissue.

**Fig. 3:**
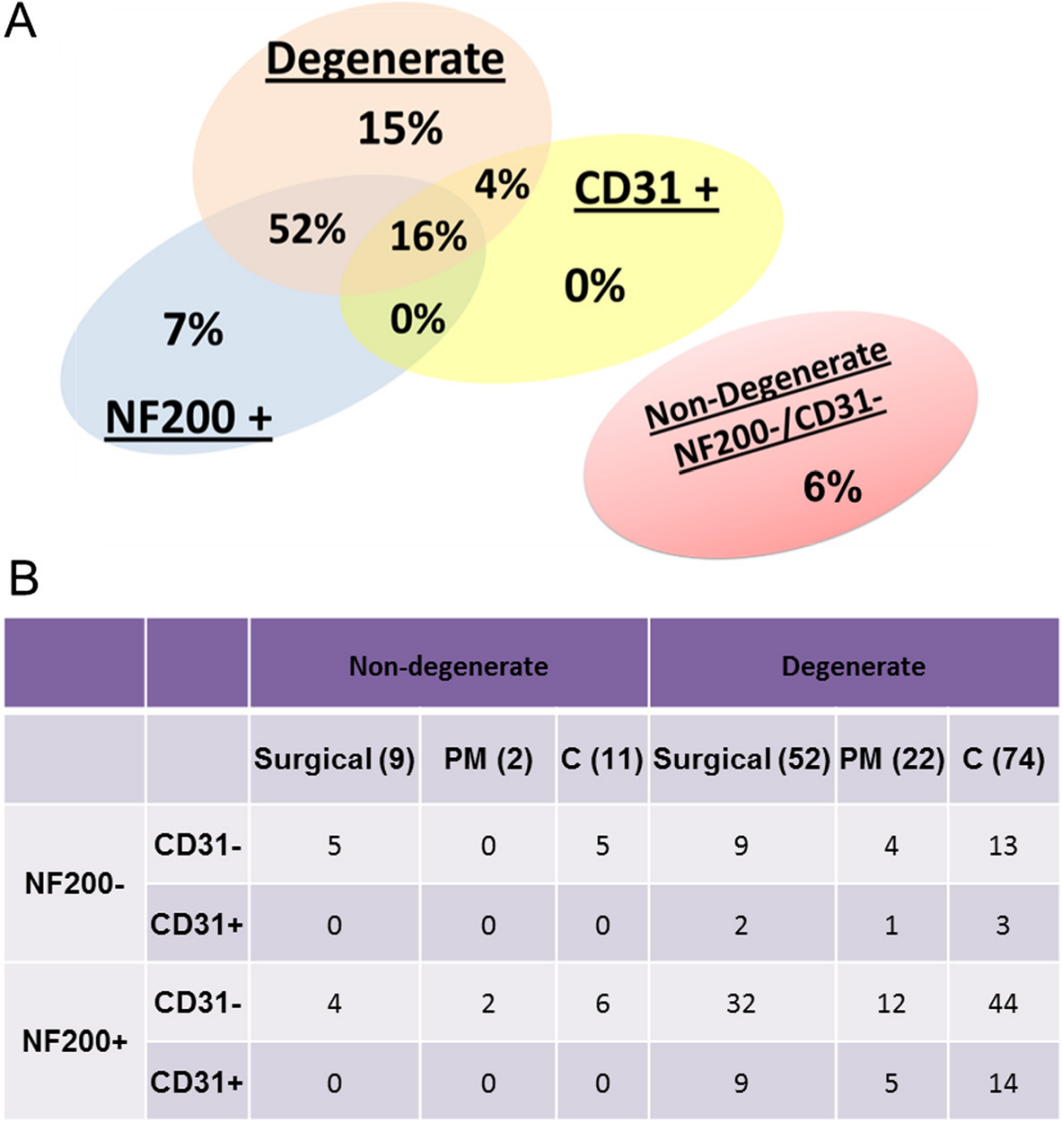
Immunopositivity of neurofilament 200 (NF200) and cluster of differentiation factor 31 (CD31) in human intervertebral disc (IVD) tissues. **a** Overall percentage of human IVD tissues where nerves and blood vessels were present or absent. **b** Number of tissues immunopositive for NF200 and/or CD31 in the non-degenerate and degenerate cohorts within post-mortem (PM) and surgical samples

Tissues investigated for the presence of nerves (NF200) and blood vessels (CD31) were separated on the basis of grade of degeneration and whether they were positive or negative for nerves and/or blood vessels (Table 1, Fig. 3). Analysis revealed that 15 % of all samples were degenerate and negative for nerves and blood vessels (NF200−/CD31−), whilst 16 % of all samples were degenerate with nerves and blood vessels (NF200+/CD31+) (Fig. 3a). In the largest cohort, we identified 52 % of samples that were degenerate with nerves but no blood vessels (NF200+/CD31−) (Fig. 3a). Interestingly, only 4 % of all samples were degenerate with no nerves but positive for blood vessels (NF200−/CD31+) (Fig. 3a). Of the 85 samples investigated, only 6 % were non-degenerate without nerves and blood vessels (NF200−/CD31−) and 7 % of non-degenerate discs had nerves but no blood vessels (NF200+/CD31−) (Fig. 3a). No samples were identified as being non-degenerate with nerves but no blood vessels (NF200+/CD31−) or non-degenerate with no nerves and positive for blood vessels (NF200−/CD31+) (Fig. 3a). No differences were seen in the proportions of each group between PM and surgical samples (Fig. 3b). Statistical analysis revealed a significant difference in the number of tissue samples displaying nerves and blood vessels between degenerate and non-degenerate cohorts (*p* < 0.0001) (Fig. 3b). Proportionality tests were performed to detect any significant differences between the proportion of samples with nerves and blood vessels. The proportion of degenerate samples with nerves was significantly higher than the proportion of non-degenerate samples with nerves present (*p* = 0.0430). The proportion of degenerate samples expressing CD31 was significantly higher than within the non-degenerate cohort (*p* = 0.0378). Non-degenerate and degenerate samples demonstrated significantly higher proportions of samples expressing nerves as opposed to blood vessels (*p* = 0.0156 and *p* < 0.0001, respectively) (Fig. 3b).

## Discussion

This study addresses the controversial topic of nerve and blood vessel ingrowth into the IVD in a large number of patient samples obtained from surgery and PM. The data presented show that nerves are present within the IAF and NP of degenerate IVDs from patients with histological evidence of disc degeneration, which confirms results shown in previous studies [21, 22, 27–30]. Studies by Freemont et al*.* demonstrated a link between nerve ingrowth and painful discs in a cohort of 80 patient samples [21]. Their study and a similar study by Brown et al. demonstrated that ingrowing nerves synthesised small reactive substances involved in nociception, such as substance P and CGRP [29], which could sensitise nerves to painful stimuli. Freemont et al. furthered their investigation by demonstrating that nerves entering through the vertebral endplates were co-localised to nerve growth factor (NGF)–expressing blood vessels [28], forming the hypothesis that endothelial cells enter the IVD first, followed by NGF receptor–expressing nerves which track alongside the endothelial cells. Coppes et al. also identified substance P–expressing nerve fibres extending into the IAF of surgically removed IVD tissue [30].

More recent studies have shown that NP cells are able to produce sensory peptides. Substance P and CGRP and their regulation can be upregulated upon cytokine stimulation [13]. Hence, if there is an increase in the production of factors involved in sensitisation of nerve endings, mechanical stimuli which are usually innocuous to the disc nociceptors could in some cases cause amplified pain sensation.

A possible initiating factor of IVD degeneration is the disruption of the vertebral endplate. Ohtori et al. observed strong correlations between modic changes and innervation within the endplate and demonstrated links between increased tumour necrosis factor (TNF)-α production and nerve ingrowth [27]. Endplate disruption is also thought to result in increased catabolic enzyme production, which can degrade matrix molecules such as aggrecan. Aggrecan derived from human IVDs is known for its ability to inhibit neurite outgrowth and endothelial cell invasion [17, 18]. Catabolic effects on the IVD, in particular the loss of proteoglycans, has been suggested to be mediated by growth factors and cytokines, in particular interleukin (IL)-1β and TNF-α. Le Maitre et al. identified increased expression of matrix metalloproteinase (MMP)-1, MMP-3 and MMP-13, as well as a disintegrin and metalloproteinase with thrombospondin motifs 4 (ADAMTS4), from human NP cells in degenerate samples compared with non-degenerate samples [31]. Following from this, later studies by the same group demonstrated regulation of MMP-3, MMP-13 and ADAMTS4 by IL-1β in human NP cells [25, 32, 33]. The loss of proteoglycans via the upregulation of such aggrecanases from human NP cells could permit the entry of stray nerves and blood vessels. Aggrecan is also extensively known for its effect on disc hydration by imbibing water; therefore, the cleavage of aggrecan in degeneration can lead to loss of disc height and pressure alterations within the NP, which can lead to the extensive fissuring seen in degenerate IVDs [22]. Nerves found within IVDs investigated in the present study are predominantly found close to fissures which may provide a route into the inner areas of the IVD where the fibrous matrix is disrupted. This is in agreement with studies by Stefanakis et al., who reported strong localisation of nerve and blood vessels to annular fissures due to its conducive environment to nerves and blood vessels [22], hence providing a likely route into the deeper layers of the IVD. Brown et al. also demonstrated that in severely degenerate IVDs, where disc height is largely compromised, there is an increase in the amount of blood vessels present around the endplate [29], which ties in with the loss of proteoglycans and water.

Unlike other researchers who have found strong co-localisation of nerves with blood vessels [28, 29, 34, 35], we observed nerve association with blood vessels on only a few occasions. Subsequent analysis of the IVD tissues revealed no samples which were histologically graded as non-degenerate that had blood vessels and no nerves. The current hypothesis that surrounds nerve and blood vessel ingrowth into the IVD is based upon NGF-expressing endothelial cells entering the IVD first, followed by sensory nerve fibres tracking alongside. In this study, we did not find this to be the case; we observed the presence of nerves without blood vessels within both surgical and PM samples. Both surgical and PM samples followed similar trends in terms of the proportions of samples expressing nerve and blood vessels, thus suggesting there is no difference between processing techniques or section thickness. Filamentous staining was observed more often in the surgical samples than in the PM samples, which is thought to be due to the thicker sections used in these samples. However, it has to be taken into account that we looked at one section of the sample and therefore cannot conclude that the sample does not have nerves and/or blood vessels in subsequent sections. Ideally, the whole tissue sample would have been sectioned serially, allowing observation of the whole specimen rather than one section. Due to the nature of the tissue received from surgery, it is difficult to determine where the nerves and blood vessels are derived from, whether it is through the endplate or the AF.

Many studies have used PGP9.5 as a marker of choice for the identification of nerves within IVD tissue [16, 20, 27, 34, 36–38]. However, in the present study, NP cell immunopositivity was observed in many tissues. This is interesting because no other studies have demonstrated NP cells being immunopositive for this neural marker.

Studies using polymerase chain reactions to identify PGP9.5 in cells isolated from human NP are quite contradictory. Lee et al. demonstrated a strong positive correlation with expression levels of PGP9.5 and NGF within NP cells [16], whereas Tolofari et al. observed no expression of PGP9.5 within human NP cells from varying grades of degeneration [20]. In studies using immunohistochemistry to identify nerve fibres within the IVD, researchers described their staining as specific to nerve fibres and did not mention any PGP9.5-immunopositive NP cells [16, 20, 27, 29, 35, 39], although no images containing NP cells were published in their reports.

## Conclusions

This study demonstrates a possible link between nerve ingrowth and degeneration of the IVD. It importantly identifies potential nerve ingrowth in the absence of blood vessels, suggesting that nerves do not follow blood vessels as previously suggested. However, further research should be carried out to investigate the direction of ingrowth.

## Abbreviations

ADAMTS4: a disintegrin and metalloproteinase with thrombospondin motifs 4
AF: annulus fibrosus
BSA: bovine serum albumin
CD31: cluster of differentiation factor 31
CEP: cartilaginous endplate
CGRP: calcitonin gene-related peptide
DRG: dorsal root ganglion
IAF: inner annulus fibrosus
IL: interleukin
IVD: intervertebral disc
LBP: low back pain
MMP: matrix metalloproteinase
ND: non-degenerate
NF200: neurofilament 200
NGF: nerve growth factor
NP: nucleus pulposus
OAF: outer annulus fibrosus
PGP: protein gene product
PM: post-mortem
SHU: Sheffield Hallam University
TBS: Tris-buffered saline
TNF: tumour necrosis factor
UMC: University Medical Centre
UT: Utrecht
